# Quinine-Induced Disseminated Intravascular Coagulation

**DOI:** 10.1155/2016/9136825

**Published:** 2016-05-16

**Authors:** Firas Abed, Ramkaji Baniya, Ghassan Bachuwa

**Affiliations:** ^1^Hurley Medical Center, College of Human Medicine, Michigan State University, Flint, MI, USA; ^2^Henry Ford Hospital, Detroit, MI, USA

## Abstract

Every drug comes with some side effect. It is the benefit/risk ratio that determines the medical use of the drug. Quinine, a known antimalarial drug, has been used for nocturnal leg cramps since the 1930s; it is associated with severe life-threatening hematological and cardiovascular side effects. Disseminated intravascular coagulation (DIC), albeit rare, is a known coagulopathy associated with Quinine. It is imperative to inquire about the Quinine intake in medication history in patients with coagulopathy, as most patients still consider it a harmless home remedy for nocturnal leg cramps. In this report, we present a case of coagulopathy in a middle-aged woman, who gave a history of taking Quinine for nocturnal leg cramps, as her home remedy. Early identification of the offending agent led to the diagnosis, prompt discontinuation of the medication, and complete recovery and prevented the future possibility of recurrence.

## 1. Introduction

Quinine has been used widely for idiopathic leg cramps since the 1930s [1, 2]. However, it has been implicated in some grave and life-threatening adverse events, such as thrombocytopenia, hypersensitivity reaction, and prolonged QT interval on electrocardiography [3]. Currently in the United States, Quinine is a prescription medication, approved by the Food and Drug Administration (FDA), solely, to be used for the treatment of uncomplicated malaria, caused by the parasite* Plasmodium falciparum*, primarily, in travellers returning from the malaria-endemic areas [4]. Despite the warning against the off-labelled use of Quinine, the FDA estimated some 51,800 patients receiving dispensed prescription for Quinine from the US outpatient retail pharmacies during the year 2011 only. So, the serious adverse effects may still continue to be reported [3]. In this corresponding study, we are reporting a case of a 45-year-old woman, who developed disseminated intravascular coagulation (DIC), after taking Quinine pills for night cramps.

## 2. Case Presentation

A 45-year-old female, with a prior medical history of essential hypertension, anxiety, and IgA nephropathy with a normal renal function, presented to the emergency department with palpitations, light-headedness, abdominal pain, nausea, vomiting, and diarrhea.

Symptoms started about 4 hours after she took two tablets of Quinine 324 mg each, which she got from her mother for nocturnal leg cramps.

She had no cough, dysuria, hematuria, headache, hematemesis, hematochezia, or melena.

She is taking amlodipine for hypertension and alprazolam for anxiety, and there is no recent change in her medications.

She quit smoking about 15 years ago; she denies alcohol or illicit drugs use.

There was no family history of hematological diseases.

Further history revealed that she had a similar episode of palpitations five months ago, after taking Quinine tablets. At that time, she was admitted to the hospital, where lab tests showed prolonged prothrombin time (PT), international normalized ratio (INR), activated partial thromboplastin time (aPTT), and elevated D-dimer level, with a normal platelet count. However, no explanation was found at that time for these findings, as nobody had linked her symptoms and lab findings to Quinine intake.

Examination revealed alert and oriented, middle-aged woman, who was not in acute distress, she had mild dehydration, heart rate was 167 beats/minute, blood pressure was 123/80 mmHg and respiratory rate was 28/minute, the temperature was 39.5 Celsius, she had no ecchymosis but had diffuse abdominal tenderness, without rebound tenderness or hepatosplenomegaly, and the rest of the general physical and systemic examination was normal.

She was admitted to the intensive care unit and treated initially as DIC possibly due to sepsis or drug induced.

### 2.1. Investigation

Electrocardiogram showed sinus tachycardia with a heart rate of 151 beats/minute.

Complete blood count, coagulation profile, and other relevant laboratory findings are given in Table 1.

With the conglomeration of many coagulation defects, prolonged PT, aPTT, increased INR, and D-dimer level, associated with decreased level of fibrinogen, in addition to anemia, leucopenia, and thrombocytopenia, all these were consistent with the diagnosis of disseminated intravascular coagulation. Peripheral blood smear was normal and no schistocytes were found. The coagulopathy worsened to nadir for the next 48 hours before showing any improvement.

Levels of liver transaminase, bilirubin, alkaline phosphatase, and thyroid function were within normal limits. Direct globulin test was positive but test for Quinine-induced platelet autoantibodies was not done. Urine toxicology screen was negative for any illicit drug.

Chest X-ray, urinalysis, and blood and urine cultures remained negative.

Antinuclear antibodies (ANA) were negative and lupus anticoagulant was undetected.

### 2.2. Differential Diagnosis

The differential diagnosis includes conditions associated with bleeding tendency, hypercoagulability, other causes of microangiopathic hemolytic anemia (MAHA), and thrombocytopenia:

- Thrombotic thrombocytopenic purpura (TTP).
- Hemolytic uremic syndrome (HUS).
- Sepsis.
- Severe liver disease.
- Autoimmune diseases like systemic lupus erythematous (SLE) and antiphospholipid syndrome.
- Certain drugs and herbal supplement adverse effect.
- Neoplastic conditions: leukemia, lymphoma, and myelodysplastic syndrome.

Coagulation tests revealed prolonged PT and aPTT with decreased fibrinogen. Unlike DIC, patients with TTP or HUS have normal coagulation testing, because microvascular thrombi in these conditions are primarily platelet-rich and fibrin-poor thrombi and are not associated with consumption coagulopathy. Besides, Patient has no neurological manifestations and renal function was normal which does not support TTP or HUS.

Although sepsis was initially suspected and the patient was started on empiric antibiotics, later it was excluded because the patient had no evidence of infection, as shown by normal chest X-ray, urinalysis, urine culture, and blood cultures.

Liver disease can be either a cause of DIC or a consequence of DIC; the patient had no history of liver disorder; liver function test remained within normal limit.

There was no recent change in the patient's medications, no reported use of new drugs (other than Quinine), and no history of over-the-counter medicine use or any herbal agents.

Antinuclear antibodies (ANA) were negative and lupus anticoagulant was undetected.

Blood film revealed no abnormal or immature cells to suspect leukemia.

### 2.3. Treatment

The patient was admitted to the critical care unit and treated conservatively. Platelet count, coagulation profile, basic metabolic profile, and electrolytes were monitored daily. Offending agent (Quinine) was stopped promptly; she responded well to supportive management with intravenous hydration with isotonic saline and electrolyte replacement. Broad spectrum antibiotics were started initially for suspected sepsis but were discontinued after we obtained negative results of blood and urine cultures. Platelets count, PT, INR, and aPTT became within normal limit in five days of admission. No fresh frozen plasma or cryoprecipitate was required.

The patient became stable and asymptomatic with supportive treatment; she was discharged in stable condition on the sixth day of admission. She was strongly advised to avoid Quinine intake, in the form of tablets or Quinine containing beverages or herbs.

A six-week follow-up visit to the hematology clinic showed no evidence of any coagulopathy in her follow-up blood tests (Table 1).

## 3. Discussion

For many decades, Quinine remained the mainstay of treatment for nocturnal leg cramps [5–7], and various trials were done to prove its benefit [8–10]. There are low quality evidence that quinine (200–500 mg/daily) reduces number of leg cramps per day and moderate quality evidence that quinine reduces cramp intensity [11]. It is believed that it decreases the excitability of the motor end-plate, thereby reducing the muscle contractility [12]. Quinine has been implicated for some of the grave and life-threatening adverse events such as thrombocytopenia, hypersensitivity reaction, and QT interval prolongation [2]. Because it was in use for decades, prescribers underestimated its side effects. A review of reports submitted to FDA's Adverse Event Reporting System (AERS) from April 2005 to October 2008 showed 38 US cases of serious adverse events associated with Quinine use. The majority of patients (25) were taking Quinine to prevent or treat leg cramps or restless leg syndrome; only one patient was taking Quinine for the treatment of malaria [13, 14]. Among the 38 reported cases of serious adverse events, 24 were hematological, 4 cardiovascular, and 10 miscellaneous adverse reactions. The reported hematological effects were thrombocytopenia, bleeding, TTP, and drug-induced thrombocytopenia. The FDA, thus, concluded that the risks of Quinine outweighed any possible benefit for off-label use and ordered a stop to marketing of this drug for prevention or treatment of nocturnal leg cramps [15].

In a systematic review of literature by Liles et al. [16], Quinine is a common cause of drug-induced thrombocytopenia and the most common cause of drug-induced thrombotic microangiopathy. Other Quinine-induced systemic disorders have been described, which include both immune-mediated and toxic adverse reactions. One hundred and fourteen articles described 142 patients with definite or probable evidence for a causal association of Quinine with acute, immune-mediated reactions. These reactions included chills, fever, hypotension, painful acral cyanosis, disseminated intravascular coagulation, hemolytic anemia, thrombocytopenia, neutropenia, acute kidney injury, rhabdomyolysis, liver toxicity, cardiac ischemia, respiratory failure, hypoglycemia, blindness, and toxic epidermal necrolysis. One hundred and two (72%) reactions were caused by Quinine pills, 28 (20%) by Quinine-containing beverages, and 12 (8%) by five other types of exposures. Excluding 41 patients who had only dermatologic reactions, 92 (91%) of 101 patients had required hospitalization for severe illness; 30 required renal replacement therapy; three died. Quinine, even with only minute exposure from common beverages, can cause severe adverse reactions involving multiple organ systems [16].

Quinine triggers an immune response in susceptible patients which results in the production of antiplatelet antibodies, as well as antibodies against leukocytes, erythrocytes, and endothelial cells, which explains the DIC, thrombocytopenia, and coagulopathy in such cases [17].

It is imperative to ask specific questions to elicit the history of Quinine intake in such patients and to remember that Quinine tablets are not the only source of Quinine for adverse reactions. Quinine-containing beverages are also a common source.

The prompt treatment of Quinine-induced DIC, with early identification and discontinuation of offending drug, offers an excellent prognosis compared to other adult forms of coagulopathy [17].

### 3.1. Learning Points

The conclusions of this study are as follows:

- Quinine is a commonly used remedy for nocturnal leg cramps, in spite of causing a potential life-threatening coagulopathy such as DIC and thrombocytopenia.
- Disseminated intravascular coagulation is a rare hematological complication of Quinine, which should always be remembered in cases of coagulopathy and DIC, with no clear-cut precipitating etiology.
- In DIC cases, specific questioning regarding Quinine use is required, as it is believed to be a harmless and efficient leg cramps remedy by the patients. There are over-the-counter preparations and herbal supplements that contain Quinine. Affected patients should be advised never to take Quinine in any form including bitter soft drinks or tonic.
- Once diagnosed, prompt discontinuation of Quinine offers an excellent prognosis of Quinine-induced DIC, compared to other adult forms of coagulopathy and DIC.
- Physician's and community awareness is essential, regarding the adverse effects associated with Quinine use. It is still prescribed in the USA, despite FDA's warning against off-label use. It should be emphasized that even small quantity of Quinine present in some beverages may trigger severe or even life-threatening side effect.

## Figures and Tables

**Table 1:** Lab results on admission and most deranged value at discharge and six weeks after discharge.

Laboratory test (ref range & units)	On admission	Most deranged value during admission	At discharge	At 6 weeks after discharge
White blood cells (4.0–10.8 K/UL)	2.1 × 10^9^ cells/L	2.1 × 10^9^ cells/L	6.3 × 10^9^ cells/L	6.1 × 10^9^ cells/L

Haemoglobin (12.0–16.0 G/DL)	15.8 gm/dL	11.2 gm/dL	11.9 gm/dL	13.0 gm/dL

Platelets (130–430 K/UL)	233 × 10^9^ cells/L	8.1 × 10^9^ cells/L	130 × 10^9^ cells/L	268 × 10^9^ cells/L

Neutrophil (36–75%)	35%	35%	58%	58%

Lymphocyte (20–50%)	34%	2%	32%	30%

Blood urea nitrogen (6–20 MG/DL)	21 mg/dL	21 mg/dL	8 mg/dL	14 mg/dL

Creatinine (0.5–1.1 MG/DL)	1.2 mg/dL	1.0 mg/dL	1.0 mg/dL	1.0 mg/dL

Prothrombin time (PT) (12.0–14.7 seconds)	19.4 seconds	25.5 seconds	14.3 seconds	12.9 seconds

International normalized ratio (INR) (<4.0)	1.68	2.38	1.33	0.99

Partial thromboplastin time (aPTT) (23.3–35.7 seconds)	49.9 seconds	76.5 seconds	25.6 seconds	27.7 seconds

D-dimer level (0–499 NG/ML)	>10,000.00 ng/dL	—	—	—

Fibrinogen (193–473 MG)	69 mg	69 mg	291 mg	359 mg

Lactate dehydrogenase (100–225 U/L)	584 U/L	—	—	145 U/L

Haptoglobin (16–200 MG/DL)	5.8 mg/dL	—	—	—

Reticulocytes (0.5–2.0%)	3.88%	—	—	—
